# Imbalance Fault Detection Based on the Integrated Analysis Strategy for Marine Current Turbines under Variable Current Speed

**DOI:** 10.3390/e22101069

**Published:** 2020-09-24

**Authors:** Tao Xie, Tianzhen Wang, Demba Diallo, Hubert Razik

**Affiliations:** 1 Logistics Engineering College, Shanghai Maritime University, Shanghai 201306, China; 201840210002@stu.shmtu.edu.cn (T.X.); demba.diallo@geeps.centralesupelec.fr (D.D.); hubert.razik@univ-lyon1.fr (H.R.); 2 CentraleSupelec, Universite Paris-Saclay, CNRS, GeePs, 3-11 Rue Joliot Curie, 91192 Gif/Yvette, France; 3 Laboratory Ampère UMR 5005, University of Lyon, 69007 Lyon, France

**Keywords:** marine current turbine (MCT), Concordia transform (CT), adaptive proportional sampling frequency (APSF), imbalance fault

## Abstract

The conversion of marine current energy into electricity with marine current turbines (MCTs) promises renewable energy. However, the reliability and power quality of marine current turbines are degraded due to marine biological attachments on the blades. To benefit from all the information embedded in the three phases, we created a fault feature that was the derivative of the current vector modulus in a Concordia reference frame. Moreover, because of the varying marine current speed, fault features were non-stationary. A transformation based on new adaptive proportional sampling frequency (APSF) transformed them into stationary ones. The fault indicator was derived from the amplitude of the shaft rotating frequency, which was itself derived from its power spectrum. The method was validated with data collected from a test bed composed of a marine current turbine coupled to a 230 W permanent magnet synchronous generator. The results showed the efficiency of the method to detect an introduced imbalance fault with an additional mass of 80–220 g attached to blades. In comparison to methods that use a single piece of electrical information (phase current or voltage), the fault indicator based on the three currents was found to be, on average, 2.2 times greater. The results also showed that the fault indicator increased monotonically with the fault severity, with a 1.8 times-higher variation rate, as well as that the method is robust for the flow current speed that varies from 0.95 to 1.3 m/s.

## 1. Introduction

Environmental concerns have contributed to the development of the exploitation of marine energies to produce electrical energy using marine current turbines (MCTs) [1,2]. Compared with wind turbines (WTs), MCTs are subject to more harsh operating conditions in the ocean. These conditions are likely to encourage the occurrence of failures [3,4,5], among which mechanical ones are the most frequent [6]. The detection of this kind of fault is delicate because of the frequent variation in the speed of sea currents. Indeed, this variation changes the rotating frequency of a turbine [7,8,9]. Therefore, it is relevant to study how to extract imbalance fault features under variable velocity conditions to maintain the safe and stable operation of the MCTs.

Accelerometers and cameras have been used for MCT fault detection and diagnosis. Signal information processing techniques are used to extract fault features from vibration signals and images [10,11,12,13]. Xia et al. [14] applied a modified convolutional neural network method to classify the bearing fault types. However, these methods require additional external equipment that must be installed in a harsh environment and may be in direct contact with seawater. Therefore, the cost of not only installation but also maintenance is high, as this equipment is prone to failure.

Therefore, the use of in-built sensors is highly preferable. Generator current sensors are already available for control purposes. Hence, motor stator current analysis (MCSA) is very prevalent for fault detection. A grey-box modelling technique for studying the swirl characteristics of gas turbine combustion systems was developed in [15] by Zhang et al. The program successfully detected the compound faults of a gas turbine based on a temperature profile. Meanwhile, the speed of the marine current is complicated and changeable, which makes it difficult to establish a complete model of a marine current power generation system. Li et al. [16] used a derivative method to highlight WTs’ blade imbalance fault characteristics. Zhang et al. [17], Feng et al. [18], and Deng et al. [19] applied a Hilbert transform (HT) to retrieve an instantaneous frequency (IF) from which fault characteristics could be derived. Salameh et al. [20] and Amirat et al. [21] proposed a method based on empirical mode decomposition (EMD) filtering to demodulate generator stator current. The EMD method can reduce the disturbance information in a stator current signal due to turbulence and waves. Gong et al. proposed a new resampling method in [22,23] called the shaft rotating frequency (1P) invariant method that can retrieve a fault frequency under the condition of varying wind speed, but there is no criterion for determining the objective time indexes and stopping iteration. An imbalance fault indicator of wind turbine blade based on the Park’s vector transforms was presented in [24]—however, variable wind speed was not considered. The d–q coordinate transformation method was used to find the fault features by Sheng et al. [25] based on a position estimator. The square of the open-loop stator current was used for blade fault detection Pires et al. in [26], but it required an efficient denoising method. Instantaneous power signal and electromagnetic torque signals were analyzed to extract blade imbalance fault characteristics (Xin et al. [27] and Xu et al. [28]). The method proposed by Tang et al. [29] used WT (wavelet transform) to filter out the supply frequency of an MCT current; however, the tuning of the wavelet transform with a variable current flow was still unaddressed.

All the above studies rarely considered that the stator current exhibits a strong interference because of variable sea current speeds. The method proposed in this work takes advantage of the three-phase system and exploits all the available information. Hence, an adaptive frequency proportional sampling method was combined with the Concordia transform to extract the features of fault imbalance. This method does not require the phase information as a frequency proportion, and it can iteratively generate an optimal resampling rate. At first, the generator stator currents were measured and projected in a new reference frame using the Concordia transform to calculate the Concordia transform modules (CTMs). Second, to address the variable fault features, a novel adaptive proportional sampling frequency (APSF) method was proposed to obtain a stationary signal. Finally, imbalance fault detection was performed by a spectral analysis. Compared with the detection method using single-phase stator current or voltage, the proposed method can more effectively detect the imbalance fault under variable conditions.

The paper is organized as follows: In Section 2, the detection problem is described. Section 3 presents the proposed CT approach based on the APSF method. Section 4 validates the proposed method through simulation and experimental results. Section 5 concludes the paper.

## 2. MCT Imbalance Fault Description 

Marine current turbines harvest marine kinetic energy and convert it into electric power. However, due to their natural environment, power quality can be degraded due to faults like blade imbalance. Even if this fault type has a signature in the frequency domain, the fault characteristics are highly sensitive to marine current velocity. This makes fault detection more difficult.

### 2.1. Marine Current Variable Speed Effect

Though marine current is predictable and varies slowly, there are still many factors that affect the steady transformation of kinetic energy by MCTs. One of the most severe is the interaction of the marine current with waves and turbulences [30]. In this case, the mechanical torque $T_{mech}\left(t\right)$ produced by the turbine is described as:

(1) $$T_{mech}=\rho C\pi R^{2}V_{current}^{3}/2\omega_{m}$$

where *C_p_* is the power coefficient, *ρ* is the density of marine current, *R* is the diameter of the blade, *V_current_* is the marine current speed, and *ω_m_* is the shaft rotating speed that changes with marine current velocity in an extensive range. This is confirmed with Equation (2), where *λ/R* (*λ* is the blade tip speed ratio) is a constant value:

(2) $$\omega_{m}=\lambda \frac{V_{current}}{R}$$

**Figure 1:**
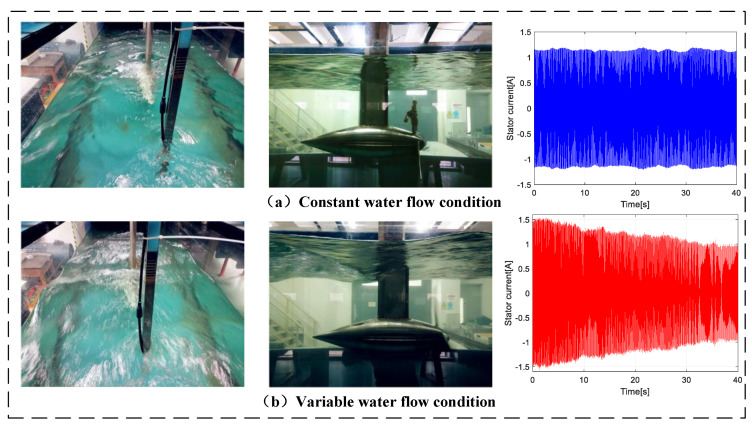
Experimental test bed and marine current turbine (MCT) stator current.

Figure 1 shows from left to right, respectively, the water basin, the MCT, and the current waveform in a stator generator. Furthermore, one can notice the different operating conditions in the top and bottom as constant and variable water flows, respectively, in the basin. Compared to Figure 1a, the stator current in Figure 1b is more disrupted and contains more interference. This clearly shows that an MCT is strongly disturbed by a variable water flow. Meanwhile, the shaft rotating frequency $f_{m}$ is variable as follows:

(3) $$f_{m}=\frac{\omega_{m}}{\left(2\pi \right)}$$

The change of the shaft rotating frequency can reveal that the frequency of the stator current signal frequently changes. This effect not only causes a change in the amplitude of the stator current signal but also brings strong interference to the instantaneous frequency, as follows:

(4) $$f_{e}=pf_{m}$$

where $f_{e}$ is the instantaneous frequency of stator current; in practice, the shaft rotation of an MCT varies greatly with variable marine currents, which means that the extracted fault characteristic frequency is variable.

### 2.2. Blade Imbalance Fault Effect

**Figure 2:**
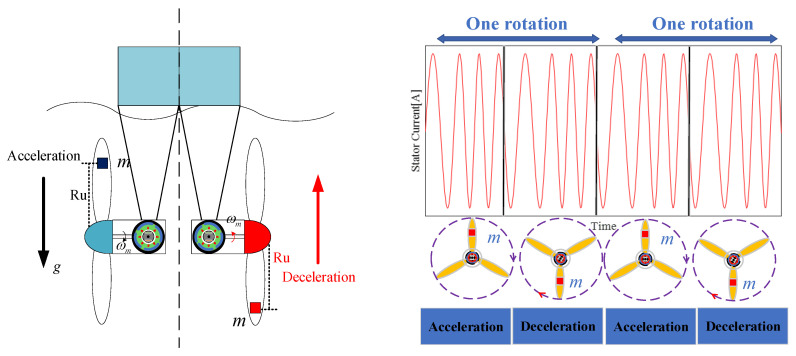
Effect of blade imbalance fault on the MCT stator current.

As critical components of energy capture, the states of blades affect the quality of power output. In addition to surges and turbulence, plankton and marine sediments settle on the blades, thus resulting in mass imbalance. This is shown in Figure 2, where *m* is the additional mass and *R_u_* is the distance between the mass and the hub. In this case, the output mechanical torque $T_{n}$ becomes:

(5) $$T_{n}=T_{mech}+T_{im}\sin\left(\omega_{m}t+\phi \right)$$

where $T_{mech}$ is the torque under healthy condition, $T_{im}=mgR_{u}$ is the amplitude of the additional torque due to mass imbalance, and ϕ is the initial phase angle.

The motion equation of the generator can be modified, as in [31,32]:

(6) $$J_{m}\frac{d\omega_{n}}{dt}=T_{n}-T_{e}-D\omega_{m}$$

where $J_{m}$ is the moment of inertia, $T_{e}$ is the electromagnetic torque, and *D* is the coefficient of friction.

By combining Equations (5) with (6), it can be deduced that the speed of the rotor under the imbalance fault condition becomes:

(7) $$\omega_{n}=\omega_{m}+\omega_{mr}\cos\left(\omega_{m}t+\phi \right)$$

where the imbalance contribution to the speed fluctuations is expressed as:

(8) $$\omega_{mr}=\frac{mgR_{u}}{J_{m}\omega_{m}}$$

The imbalance fault features in stator current signal caused by the abrupt increase and decrease of a turbine’s torque. However, during the transfer process, the imbalance fault feature is also affected by turbulence and other marine factors that are responsible of strong interferences. Combined with Equation (7), the stator current can be expressed as:

(9) $$i_{s}=A_{i}\cos\left[\omega_{e}t+Fcos\left(\omega_{m}t\right)\right]+\epsilon_{i}$$

where $A_{i}$ is the amplitude of stator current, *F* is called the imbalance modulation, $\omega_{e}$ is the rotor electrical speed, and $\epsilon_{i}$ represents the environmental noise. Since $J_{m}$ is large and the degree of imbalance fault is small, Equation (9) can be written as (the details are shown in Appendix A):

(10) $$i_{s}=A_{i}\cos\left(\omega_{e}t\right)+\frac{A_{i}F}{2}\sin\left(\omega_{e}+\omega_{m}\right)t+\frac{A_{i}F}{2}\sin\left(\omega_{e}-\omega_{m}\right)t+\epsilon_{i}$$

**Figure 3:**
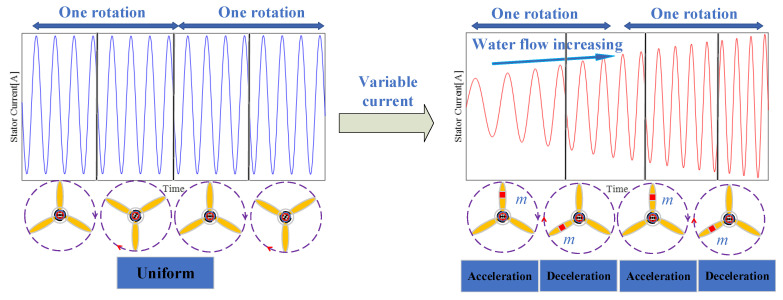
MCT mechanical rotation under different conditions.

When considering waves and variable marine currents, fault characteristics can change and be covered up by increasing water flow, as shown in Figure 3. As such, it can be observed in Figure 2 and Figure 3 when looking at the stator current waveform that the frequency changes under imbalance faults and variable water flows. Thus, demodulating the single-phase stator current can be used to get fault features, but the demodulation algorithm (e.g., the Hilbert transform) cannot accurately estimate an instantaneous frequency, thus resulting in poor fault characterization.

It can be found from Figure 3 that a single-phase current frequency itself does not contain the fault characteristics that can be found in a frequency with a changing rate. 

Single-phase voltage also contains strong interference and can be expressed as follows:

(11) $$u_{s}=A_{u}\cos\left(\omega_{e}t+\varphi \right)+\frac{A_{u}F}{2}\sin[\left(\omega_{e}+\omega_{m}\right)t+\varphi ]+\frac{A_{u}F}{2}\sin[\left(\omega_{e}-\omega_{m}\right)t+\varphi ]+\epsilon_{u}$$

where $A_{u}$ is the amplitude of stator voltage and $\epsilon_{u}$ represents the environmental noise. If the frequency rate of change is used as a fault indicator, a denoising process is required. Additionally, due to the high-intensity changes of water flow, the denoising algorithm must adjust itself. 

## 3. Fault Detection Using Concordia Transform

### 3.1. Fault Feature Extraction Based on Concordia Transform

According to Equation (10), when an imbalance fault occurs, the three-phase stator currents can be expressed as:

(12) $$i_{a}=I_{f\;}\cos\left(\omega_{e}t-\alpha \right)+\;I_{dl}\cos\left(\left(\omega_{e}-\omega_{m}\right)t-\beta_{l}\right)+I_{dr}\cos\left(\left(\omega_{e}+\omega_{m}\right)t-\beta_{r}\right)$$

(13) $$i_{b}=I_{f\;}\cos\left(\omega_{e}t-\alpha -\frac{2\pi }{3}\right)+I_{dl}\cos\left(\left(\omega_{e}-\omega_{m}\right)t-\beta_{l}-\frac{2\pi }{3}\right)+I_{dr}\cos\left(\left(\omega_{e}+\omega_{m}\right)t-\beta_{r}-\frac{2\pi }{3}\right)$$

(14) $$i_{c}=I_{f\;}\cos\left(\omega_{e}t-\alpha +\frac{2\pi }{3}\right)+I_{dl}\cos\left(\left(\omega_{e}-\omega_{m}\right)t-\beta_{l}+\frac{2\pi }{3}\right)+I_{dr}\cos\left(\left(\omega_{e}+\omega_{m}\right)t-\beta_{r}+\frac{2\pi }{3}\right)$$

where $I_{f}$ is the maximum value of the fundamental component, $I_{dl}$ is the maximum value of the current lower sideband component at the frequency $f_{e}-f_{m}$, and $I_{dr\;}$ is the maximum value of the current upper sideband component at the frequency $f_{e}+f_{m}$.

An imbalance fault can be detected by using a sideband component with just a single-phase current. However, at the early stage of a fault, the small amplitude of the harmonic components due to fault occurrence can be concealed by a fundamental component. To address this problem, the stator currents can be projected in a new reference frame using the Concordia transform. If the homopolar component is neglected, the components of the current vector are:

(15) $$i_{\alpha }=\left(2i_{a}-i_{b}-i_{c}\right)/\sqrt{6}$$

(16) $$i_{\beta }=\left(i_{b}-i_{c}\right)/\sqrt{2}$$

The module of the current is given by:

(17) $$I_{amp}={\left|i_{\alpha }+ji_{\beta }\right|}^{2}=3\left(I_{f}^{2}+I_{dl}^{2}+I_{dr}^{2}\right)/2+3I_{f}I_{dl}\cos\left(\omega_{m}t-\alpha +\beta_{l}\right)+\;3I_{f}I_{dr}\cos\left(\omega_{m}t-\alpha +\beta_{r}\right)+3I_{dl}I_{dr}\cos\left(2\omega_{m}t-\beta_{r}+\beta_{l}\right)$$

To eliminate the *DC* and the low-frequency components, the derivation of Equation (17) leads to:

(18) $$I_{damp}=-3I_{f}I_{dl}\omega_{m}\sin\left(\omega_{m}t-\alpha +\beta_{l}\right)-3I_{f}I_{dr}\omega_{m}\sin\left(\omega_{m}t-\alpha +\beta_{r}\right)\;-6I_{dl}I_{dr}\omega_{m}\sin\left(2\omega_{m}t-\beta_{r}+\beta_{l}\right)$$

It can be concluded from Equation (18) that there are only 1P and 2P-frequency components in the derivative of the modulus of the current vector. Moreover, the amplitude of the faulty component increased. It can also be observed that the component at the 2P-frequency has a lower amplitude because it does not include *I_f_*; as such, it is negligible compared to the 1P component.

Therefore, the derivative of the modulus of the current vector can be used as imbalance fault feature. It is denoted *I_damp_* in the following.

**Figure 4:**
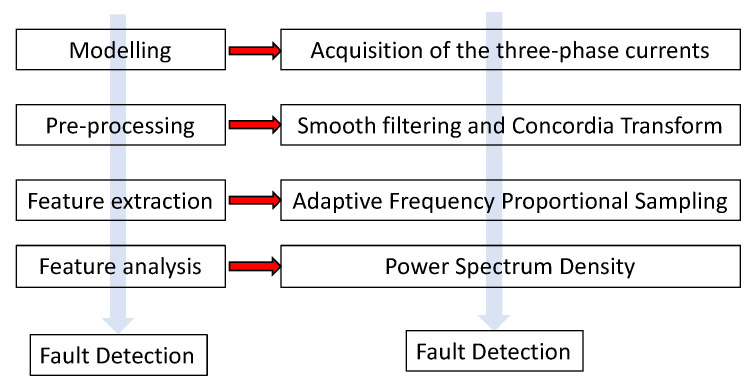
Flowchart of the fault detection method.

In conclusion, the imbalance fault detection method can be summarized in four steps as displayed in Figure 4:

- Modelling: Acquire the three-phase currents.
- Processing: Use smooth filtering and Concordia transform to calculate the components of the current vector.
- Feature extraction: Compute of the derivative of the modulus of the current vector.
- Feature analysis: Compute the power spectrum density to extract the 1P frequency and its amplitude.

In the next sections, the last two steps are detailed.

### 3.2. Feature Extraction Using an Adaptive Proportional Sampling Frequency (APSF) Method

With a uniform sampling method, the sampling frequency is constant. Therefore, the resampled shaft rotating frequency can be written as:

(19) $$f_{rs}=f_{m}⁄f_{s}$$

where *f_s_* is the sampling frequency. The frequent change of marine current velocity leads to a variable frequency of the shaft $f_{m}$. In that case, the modulus of the current vector is not processed and the fault signature is an impulse that is concealed in the frequency spectrum. However, if the sampling frequency is proportional to the shaft frequency, the characteristic frequency of the imbalance fault becomes constant and easier to detect.

#### 3.2.1. Calculate the Instantaneous Frequency Based on Zero-Crossing Estimation

According to Equation (19), the resampled shaft rotating frequency $f_{rs}$ is constant if the sampling frequency is proportional to the shaft rotating frequency. It is applied to $I_{damp}$ the signal becomes stationary. 

The key to the APSF method is how to calculate the proportional sampling frequency. This is done in three steps:

- Look for the zero-crossing point.
- Use linear interpolation to interpolate the zero-crossing sequence.
- Get the zero-crossing point series $t_{zero}\left[k\right],\;k=1,2,\ldots ,K$, and calculate the time interval between two points in a zero-crossing sequence as follows:
  (20) $$\Delta t_{zero}\left[k\right]=t_{zero}\left[k+1\right]-t_{zero}\left[k\right]$$

The short time intervals are removed by setting the threshold of small intervals to $\delta =1/f_{e\;}$ to get a new time interval sequence $\Delta t_{zero}\left[k-\delta \right]$. The instantaneous and rotation frequencies are computed from the new time interval as follows:

(21) $$f_{e}\left[k-\delta \right]=\;1/\Delta t_{zero}\left[k-\delta \right]f_{m}\left[k-\delta \right]=\;f_{e}\left[k-\delta \right]/p$$

According to the length N of the original $I_{damp}$ signal, a new N-point shaft rotating frequency sequence $f_{m}\left[n\right]$ is reconstructed by cubic spline interpolation [33].

#### 3.2.2. Iteratively Updated Proportional Frequency

The sampling frequency point can be iteratively updated to make the sampling frequency proportional to the shaft rotating frequency. A constant $f_{rs}$ can be obtained by calculating the mean of the instantaneous frequency:

(22) $$f_{rs}=\overset{N}{\underset{0}{\sum }}f_{m}\left(n\right)/N$$

The first iteration of time index is set at $S_{1}\left[1\right]=1$ and:

(23) $$S_{1}\left[n+1\right]=S_{1}\left[n\right]+f_{rs,0}/f_{m,0}\left[n\right]$$

The calculated proportional frequency indexes $S_{1}\left[n\right]$ is used to interpolate the new signal $I_{damp,1}\left[n\right]$. The updated shaft rotating frequency sequence $f_{m,1}\left[n\right]$ can be obtained by the new zero-cross point of $I_{damp,1}\left[n\right]$.

**Figure 5:**
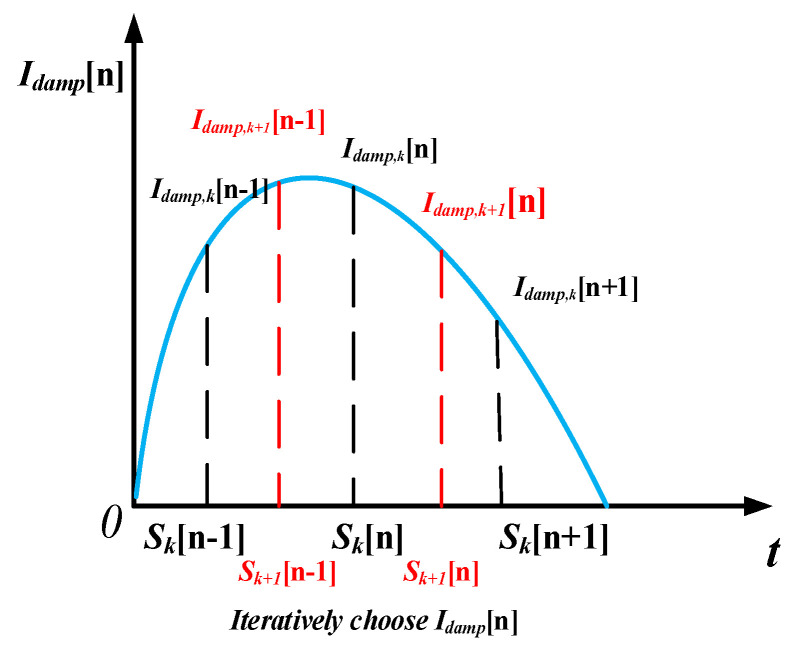
The adaptive proportional sampling frequency (APSF) method for Concordia transform modules.

As the time index is recursively updated (Figure 5), the sampling frequency becomes proportional to the shaft rotating frequency. When the resampled shaft rotating frequency $f_{rs}$ is calculated by the mean of $f_{m}\left[n\right]$, it becomes a constant value and the sampling frequency becomes proportional to the shaft rotating frequency. When the shaft rotating frequency of the MCT is high, the time step is shortened and the number of points that can be sampled point increases. Otherwise, the number of sampling points is reduced.

#### 3.2.3. Setting Criterion of Stop Iteration

The iteration is a process of continuous optimization with objective time indexes. The time series is suitable for interpolation to make the fault features constant. Figure 5 illustrates the iteration process; at the ith iteration, $I_{damp,i}\left[n\right]$ is reconstructed by cubic spline interpolation. To verify that the updated $I_{damp,i}\left[n\right]$ signal is expected, one must define the variance of the current shaft rotating frequency as:

(24) $$\sigma =\overset{N}{\underset{0}{\sum }}{\left(f_{m,i}\left(n\right)-\bar{f_{m,i}\left(n\right)}\right)}^{2}/N$$

The average value $\bar{f_{e,i}}\left(n\right)$ directly reflects the current velocity of the current period. When the current velocity increases, the data become highly volatile and difficult to be sampled as a stable signal. Therefore, the iteration process is stopped when *σ* ≤ *γ*, where γ is a threshold defined as:

(25) $$\gamma =1/\mu \bar{f_{e,i}}\left(n\right)$$

where *μ* is a regulatory factor that can be an experience value based on historical data. The threshold is set so as the fault features are more concentrated in the frequency spectrum. The setting is a trade-off between sensitivity and accuracy. A low value of *γ* is suitable for high accuracy but requires a higher sampling period, while a higher value of *γ* degrades the accuracy.

### 3.3. Fault Features Analysis

At the end of the iteration, the power spectrum density (PSD) can be applied to the fault feature $I_{damp}$ that is the derivative of the of the modulus of the current vector in the Concordia reference frame. A vector of length *L* for the sampled PSD $AP\left(f_{l}\right)$ is obtained, where *l* = 1,2,3,...,*L*. The mean value $\bar{AP}$ is defined as follows:

(26) $$\bar{AP}=\;\sum_{1}^{l}AP\left(f_{l}\right)/L$$

A fault detection indicator (FI) is defined as:

(27) $$FI=\;AP\left(f_{m}\right)/\bar{AP}$$

where $AP\left(f_{m}\right)$ is the normalized amplitude of the shaft rotating frequency component; in a healthy condition, FI is close to 1 because the normalized amplitude of the shaft rotating frequency component is close to the mean of sampled PSD. At fault occurrence, the fault indicator should be different from one.

**Figure 6:**
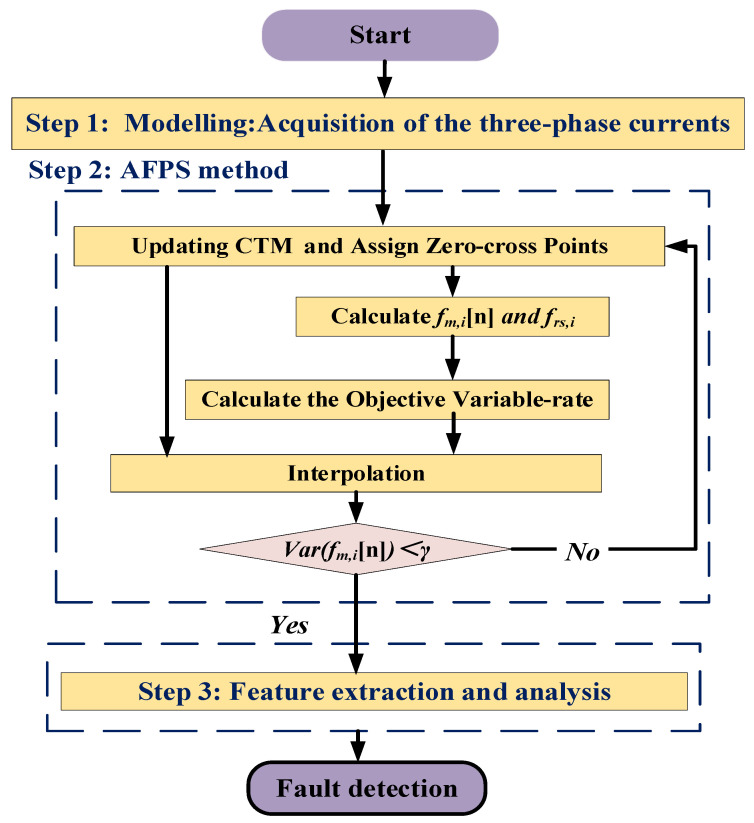
Detailed flowchart of the fault detection method.

The proposed method is finally detailed in Figure 6. It is implemented through the following steps.

(i) Acquisition of the three-current flowing into the windings of the generator: $I_{a}\left[n\right],I_{b}\left[n\right],\mathrm{and}\;I_{c}\left[n\right]$ are acquired with uniform sampling, and then the noise and strong interference are filtered through a smoothing filter.
(ii) Concordia transform: The Concordia transformation is used to transform the measured three-phase stator current signals into $I_{\alpha }\left[n\right],I_{\beta }\left[n\right]$. The derivative of the current vector modulus $I_{damp}\left[n\right]$-denoted CTM is then computed.
(iii) Obtain objective time indexes: The instantaneous frequency is iteratively calculated based on the zero-cross point method to update the proportional frequency. The objective time indexes S(n) are generated by the updated proportional frequency;
(iv) Interpolation: The samples $I_{damp,i}\left[n\right]$ are interpolated based on the objective time indexes $S_{i}\left(n\right)$ to obtain the objective CTM signal; the, $f_{m,i}\left(n\right)$ is calculated by the zero-cross point of objective CTM at each iteration.
(v) Repeat operation: Steps (iii)–(iv) are repeated until σ≤γ. The iteration is stopped, and the objective CTM is stored in $I_{damp}\left[n\right]$.
(vi) Fault feature representation: The frequency spectrum analysis is done to calculate the fault indicator.

## 4. Simulation and Experimental Results

### 4.1. Simulation Results

The simulation model included the MCT, a permanent magnet synchronous generator (PMSG), an imbalance fault generation module, and a three-phase load. To better reflect reality, the generation of wave and turbulence was added with a marine current velocity varying between 1.2 and 1.5 m/s. The sampling frequency was set at 1 kHz. Fault severities were simulated with one blade mass adjustment by +1%, +2%, and +3% (the ratio of the imbalance fault torque to the normal one), while the mass of the other two blades was constant.

**Figure 7:**
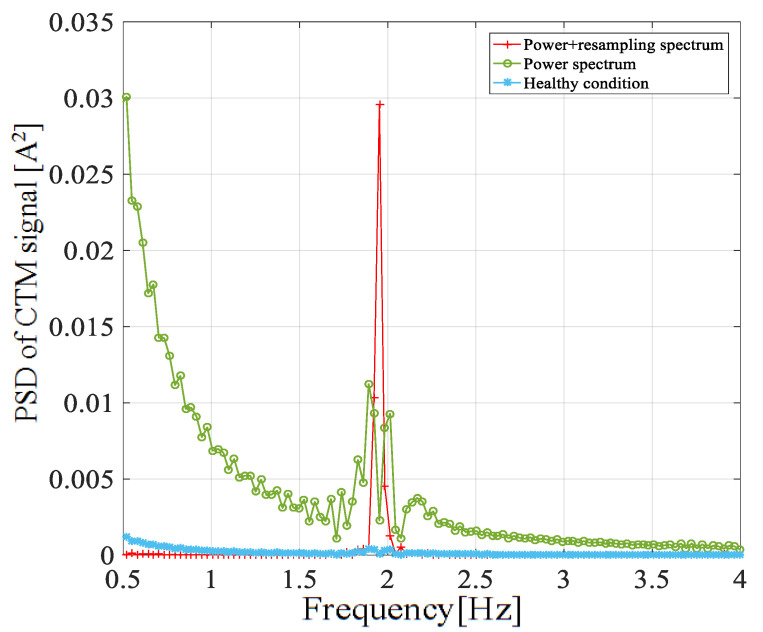
Comparison of the CTM signal in a frequency range around 1P frequency.

**Figure 8:**
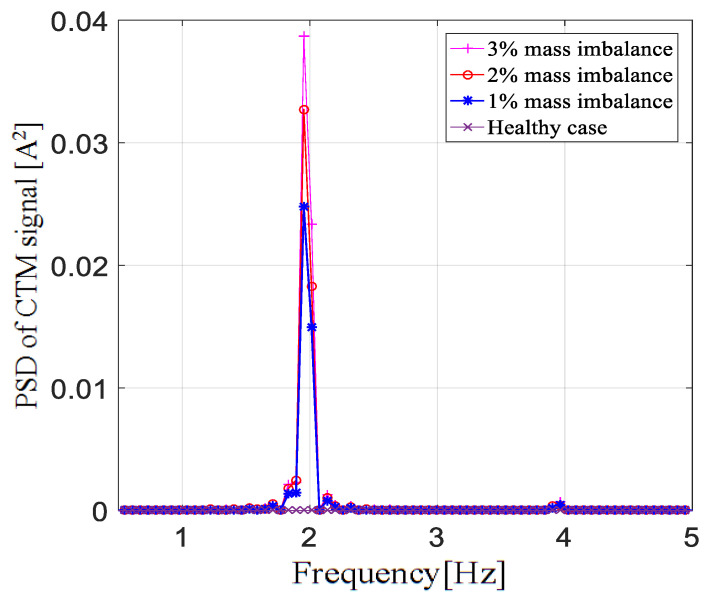
Amplitude of the 1P frequency component with different fault severities.

Figure 7 clearly shows the positive effect of the adaptive frequency proportional sampling. The fault frequency excited at 1P became more evident through the adaptive proportional frequency sampling method. Figure 8 shows the CTM spectrum under different fault severities, and it is obvious that there was no excitation at the 1P frequency in the healthy case. It can also be noted that the amplitude at 1P frequency increased as the degree of failure deepened.

Table 1 shows a comparison between the results when using a single-phase stator current or voltage with the proposed method. According to Table 1, with a +1% imbalance fault, whether the water flow rate was variable or constant, the method based on CTM had a higher sensitivity with the highest amplitude at the fault frequency.

Table 2 shows a comparison between the results when using a single-phase stator current or voltage with the proposed method under different fault severities. The following conclusions can be drawn:

1. For the lowest fault severity, the proposed method had the highest sensitivity thanks to its robustness to environmental disturbances.
2. When the fault level increased, the proposed method performed better because of the highest current amplitude.
3. Finally, the proposed indicator was proportional to the fault severity, which is of great importance for condition-based maintenance.

### 4.2. Experimental Results

**Figure 9:**
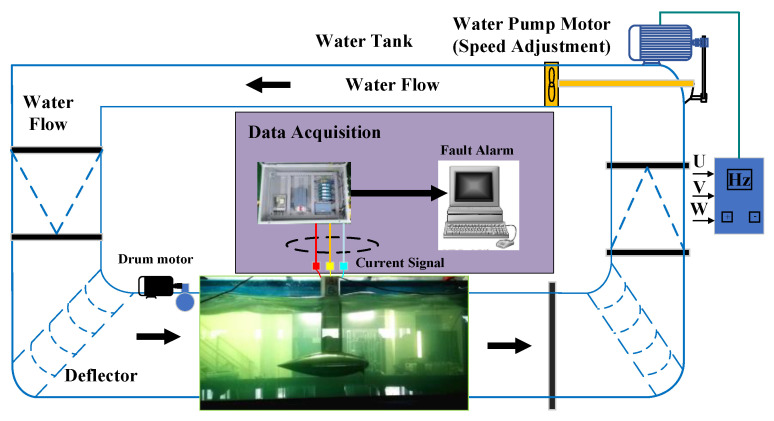
The MCT prototype with a water flow channel.

The proposed method was evaluated with data from an experimental testbed with a 230 W direct-drive PMSG. The generator is placed in the water flow channel, as shown in Figure 9. The detailed parameters of the MCT system are displayed in Table 3. The data monitoring and collection system was set up in the generator terminal. This platform could simulate stationary or non-stationary water flow, wave, and turbulence with drum and pump motors.

**Figure 10:**
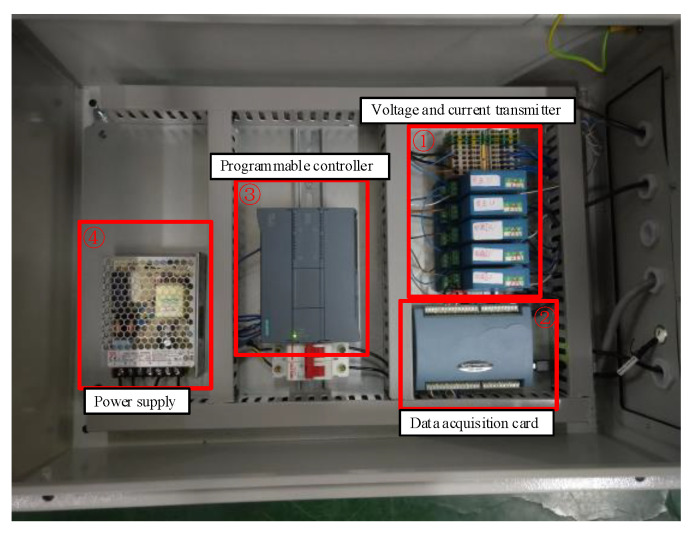
The electric data acquisition system.

The data acquisition system was composed of a power supply, a data acquisition card, a programmable controller, and a voltage (current) transmitter (as shown in Figure 10). The sampling frequency was 1 kHz. The measured data were collected and transmitted to the host computer for processing.

**Figure 11:**
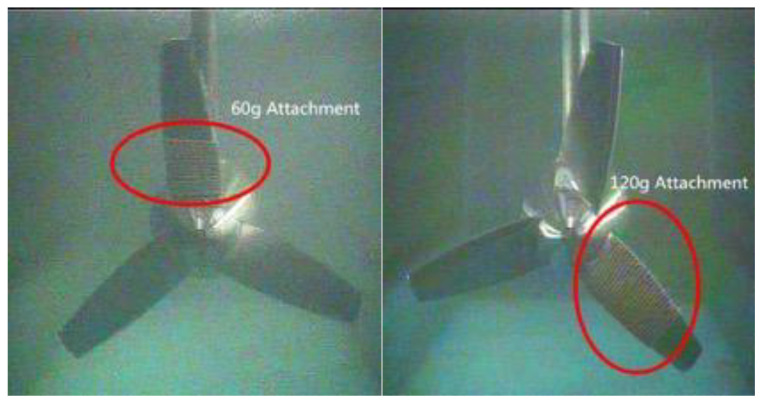
Different imbalance fault severities setting on the MCT blade.

A rope of known quality was wound around the MCT blades as an attachment to create fault severities, as shown in Figure 11. Only the mass of the one blade was changed. The pump frequency was adjusted so that the fundamental frequency did not change for the corresponding imbalanced fault test, and the load resistance was set to 50 Ω.

**Figure 12:**
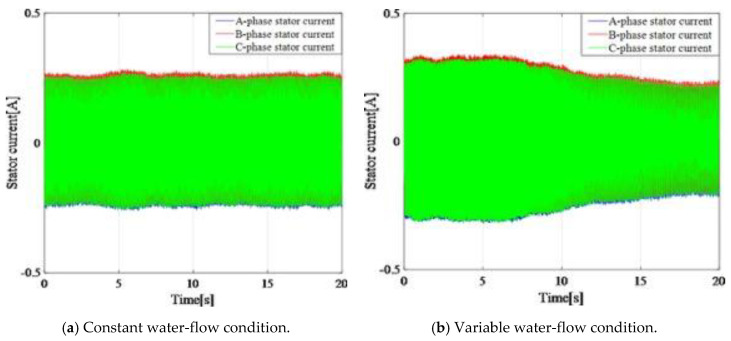
Three-phase stator currents under different water-flow conditions.

**Figure 13:**
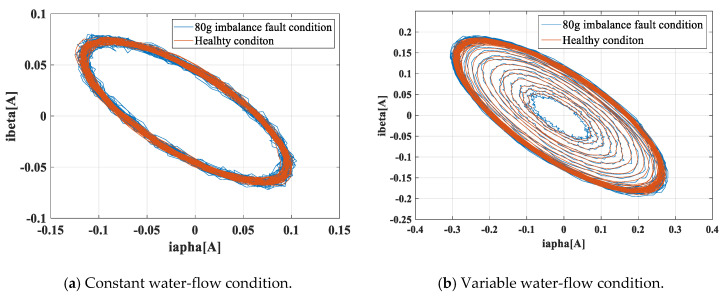
The stator currents of i_α_ and i_β_ under different water-flow conditions.

Figure 12a shows the three-phase currents collected on the experimental platform in the stationary conditions, and Figure 12b shows the three-phase currents under non-stationary conditions. It was found that the variable water flow modulated the amplitude and the frequency of the stator current, and the modulated amplitude or frequency could cover the fault features. Figure 13 shows the three currents flowing in the generator’s windings in the Concordia reference frame under stationary and non-stationary conditions. The variable conditions induced nuisances in the current vector trajectory. A fault detection method must be robust against this perturbation. 

**Figure 14:**
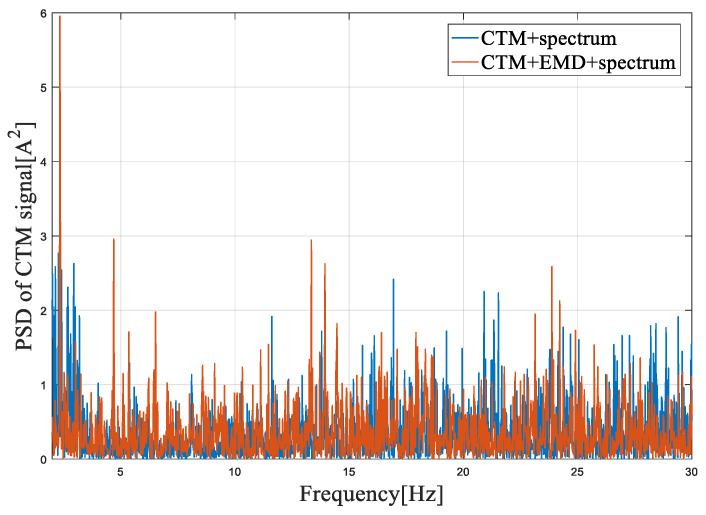
The power spectrum density (PSD) of the CTM signal in a frequency range around 1P with the empirical mode decomposition (EMD) method.

**Figure 15:**
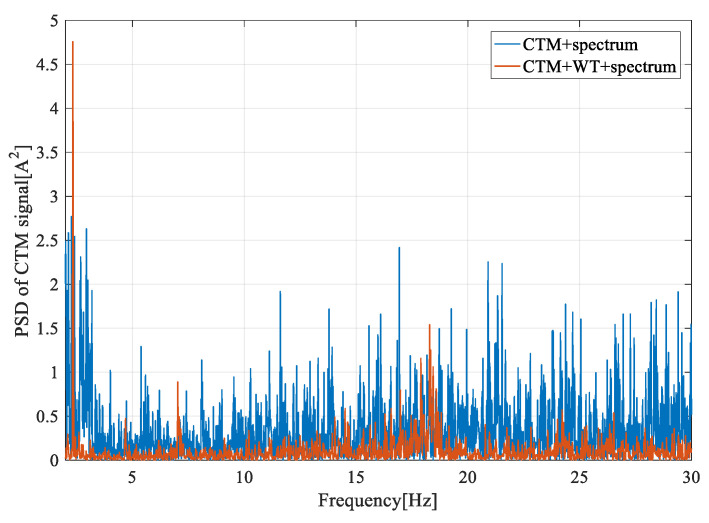
The PSD of the CTM signal in a frequency range around 1P with the wavelet transform (WT) method.

**Figure 16:**
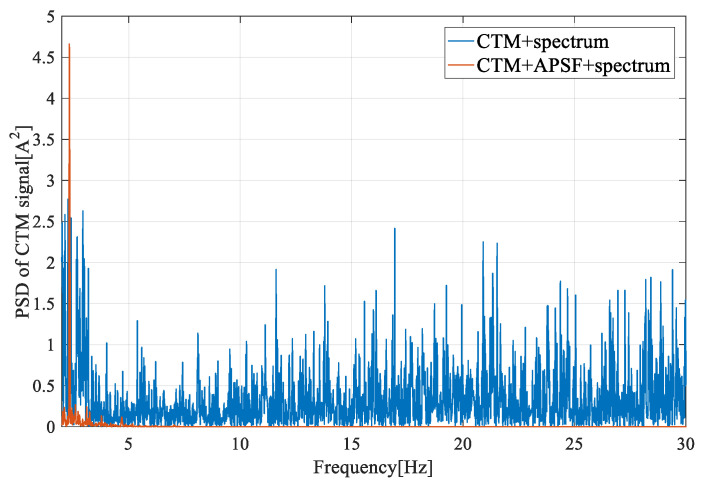
The PSD of the CTM signal in a frequency range around 1P with the APSF method.

**Figure 17:**
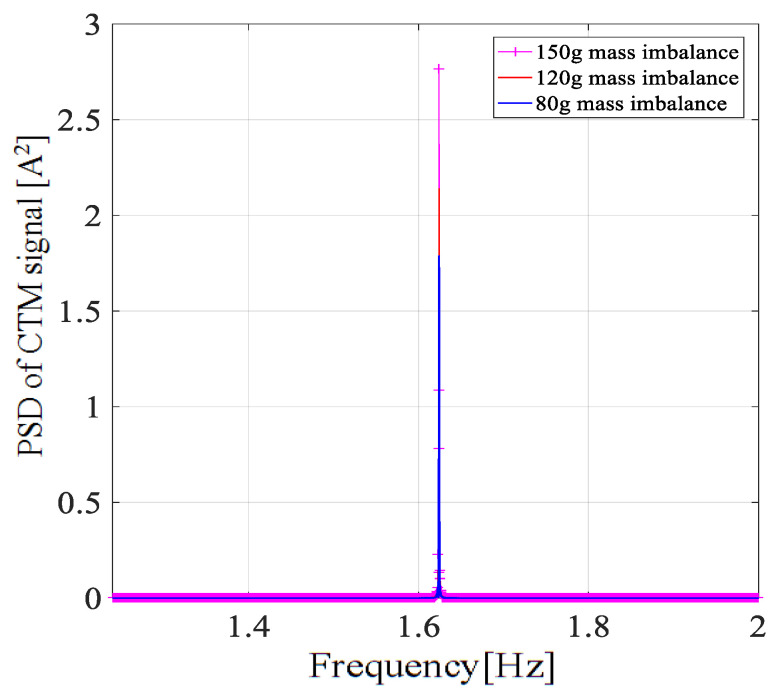
Amplitude of the 1P-frequency component with different fault severities.

Figure 14, Figure 15 and Figure 16 show the frequency spectrum, the EMD, and the WT of the same fault feature, all obtained with the proposed method. It can be observed that all three methods could manage the variable water flow. The fault characteristic frequency was stable at 1.62 Hz (fe = 12.96 Hz). However, the EMD and the WT did not filter out the supply frequency from the CTM signal, at least as performed with the proposed method. In Figure 17, only the resampling fault feature is analyzed, and the frequency spectrum for three different fault severities are plotted. With the increase of the imbalance fault degree, the fault frequency amplitude remained proportional to the fault severity, as shown in Table 4; this relationship is essential for condition-based maintenance teams.

**Figure 18:**
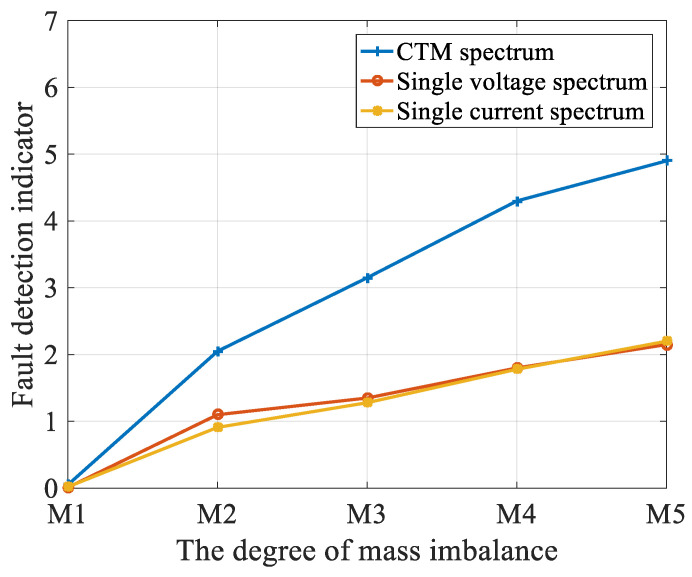
Amplitude of the spectrum under different degrees of mass imbalance. (M1: healthy case; M2: 80 g; M3: 120 g; M4: 150 g; and M5: 220 g).

Figure 18 shows the compared spectrum amplitudes for three different methods (single current, single voltage, and CTM) under a constant marine current velocity for different fault severities (all amplitudes are divided by the absolute value of the mass imbalance, if any). In the healthy condition, the fault indicator of the three methods was 0. It can be seen in Table 5 that as the degree of fault changed, the fault indicator value based on the CTM method changed to 3.71, which was higher than that of the other two methods. This implies that the resampled CTM frequency spectrum is much more sensitive to fault severity and enhances the fault feature extraction. 

**Figure 19:**
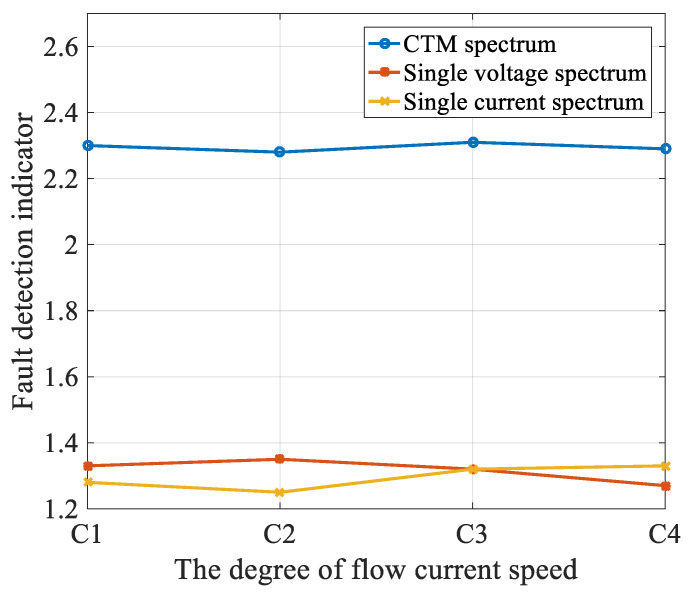
Amplitude of the spectrum under different flow current velocities. (C1: flow current velocity is 0.95 m/s; C2: 1.1 m/s; C3: 1.2 m/s; and C4: 1.3 m/s).

Figure 19 displays the spectrum amplitudes for the same three methods for different marine current speeds under a constant additional mass (M2), For all three methods, the amplitude was almost constant for all the four different flow speeds. It can be concluded that the methods were robust against variations of sea current, which could have been a nuisance.

## 5. Conclusions

This work addressed marine current turbine blade imbalance fault detection. This fault due to marine attachments induces torque and speed oscillations. The fault can be detected through an analysis of the frequency spectrum of electrical signals. However, waves and turbulences combined with the natural marine flow speed make fault detection more difficult because fault characteristics are variable and may be concealed within environmental noise. 

The methodology proposed in this paper takes the benefit of built-in current sensors and uses the three measured phase currents. The derivative of the current vector modulus in the Concordia reference frame is used as a fault feature because it is more sensitive to the 1P frequency that appears in the current spectrum during fault occurrence. The initial non-stationary signal is transformed into a stationary one thanks to an adaptive proportional frequency sampling technique. The fault indicator is based on the amplitude of the power spectrum density of the fault feature. 

Simulation and experimental results from a test bed composed of a marine current turbine coupled to a 230 W permanent magnet synchronous generator showed the efficiency of the method to detect imbalance faults. As could be seen in a comparison with the methods using a single electrical information (phase current or voltage), the fault indicator based on the three currents was, on average, 2.2 times better at detection. The experimental results also showed that the fault indicator increased monotonically with the fault severity (with an additional mass of 80–220 g attached to blades), with a 1.8 times-higher variation rate. The results also showed that the method is robust are a variable flow current speed.

## Figures and Tables

**Table 1:** Comparison of fault detection indicator (FI) values under different conditions (+1% imbalance fault). CTM: Concordia transform module.

Proposed Methods	Constant Water Speed [FI]	Variable Water Speed [FI]
With single stator current [17,18,19]	8.89	9.03
With single stator voltage	12.61	11.69
With squared stator current	10.46	8.20
With CTM	17.05	16.11

**Table 2:** Comparison of FI values under different fault severities (variable water-flow condition).

Proposed Methods	Fault 1% [FI]	Fault 2% [FI]	Fault 3% [FI]
With single stator current [17,18,19]	9.03	9.81	10.09
With single stator voltage	11.69	12.1	11.98
With squared stator current	8.20	9.2	9.97
With CTM	16.11	17.45	19.02

**Table 3:** The detailed parameters of the MCT system. PMSG: permanent magnet synchronous generator.

Turbine	Parameter	PMSG	Parameter
Water flow channel	5 × 13.5 × 2.2 m	Pole-pair	8
Current speed	0.15~2 m/s	Flux	0.18 Wb
Chord length	0.2–0.3 m	Resistance	3.3 Ω
Rotor disk diameter	0.6 m	d(q) axis inductance	11.9 mH

**Table 4:** Comparison of FI values under different fault severities (variable water-flow condition).

Proposed Methods	Fault 1 [80 g]	Fault 2 [120 g]	Fault 3 [150 g]
With CTM	1.73	2.25	2.81

**Table 5:** Comparison of FI variance under different methods.

Methods	Single Current	Single Voltage	CTM
FI Variance	0.71	0.67	3.71

## Abbreviations

MCTs—Marine current turbines	FI—Fault detection indicator
WTs—Wind turbines	EMD—Empirical mode decomposition
MCSA—Motor current signal analysis	WT—Wavelet transform
PSD—Power spectral density	HT—Hilbert transform
IF—Instantaneous frequency	IA—Instantaneous amplitude
CT—Concordia transform	APSF—Adaptive proportional sampling frequency
CTM—Concordia transform modulus	1P—Shaft rotating frequency
***Nomenclature***	
*T_n_—*Mechanical torque [N·m]	*T_0_*—Normal mechanical torque [N·m]
*J_m_—*Moment of inertia [unit]	*D*—The coefficient of friction [unit]
*R—*Diameter of the blade [m]	*C_p_—*Power coefficient [unit]
*m*—Virtual mass [kg]	*R_u_*—Distance between mass and hub [m]
*T_mech_—*Torque caused by wave [N·m]	*T_im_—*Torque caused by additional mass [N·m]
*f_m_—*Rotor mechanical frequency [Hz]	φ—Initial phase angle [rad]
*f_e_—*Supply frequency [Hz]	*g*—Gravitational acceleration [m/s]
*p—*Number of pole pair [unit]	*V_current_—*Marine current speed [m/s]
*λ—*Blade tip ratio [unit]	*ω_m_—*Rotor mechanical speed [rad/s]
*A—*The amplitude of stator current [unit]	*ε**—*Environmental noise [unit]
*i_s_—*The stator current of MCT [A]	*u_s_—*The stator voltage of MCT [V]
*I_damp_*—CTM signal series [A^2^]	*N—*Number of samples [unit]
*S—*The objective time indexes [s]	*f_s_—*Sampling frequency [Hz]
*f_rs_—*Resampled frequency [Hz]	*μ*—Regulatory factor [unit]

## Appendix A

The deduction of Equation (9) to (10) is:

(A1) $$i_{s}\left(t\right)=A_{i}\cos[\omega_{e}t+F\cos(\omega_{m}t)]+\varepsilon_{i}\left(t\right)$$

(A2) $$i_{s}\left(t\right)=A_{i}\cos(\omega_{e}t)\cos\left[F\cos(\omega_{m}t)\right]+A_{i}\sin(\omega_{e}t)\sin\left[F\cos(\omega_{m}t)\right]+\varepsilon_{i}\left(t\right)$$

Then, a lot of expressions of the Bessel function are used:

(A3) $$\cos(z\cos\omega t)=J_{0}\left(z\right)+2\overset{\infty }{\underset{p=1}{\sum }}{\left(-1\right)}^{p}J_{2p}\left(z\right)\cos2p\omega t$$

(A4) $$\cos(z\sin\omega t)=J_{0}\left(z\right)+2\overset{\infty }{\underset{p=1}{\sum }}J_{2p}\left(z\right)\cos2p\omega t$$

and

(A5) $$\sin(z\cos\omega t)=-2\overset{\infty }{\underset{p=1}{\sum }}{\left(-1\right)}^{p}J_{2p-1}\left(z\right)\cos(2p-1)\omega t$$

(A6) $$\sin(z\cos\omega t)=-2\overset{\infty }{\underset{p=1}{\sum }}J_{2p+1}\left(z\right)\cos(2p+1)\omega t$$

With $J_{n}\left(z\right)=\overset{\infty }{\underset{k=0}{\sum }}\frac{{\left(-1\right)}^{p}}{k!\left(n+k\right)!}{\left(\frac{z}{2}\right)}^{2k+n}$, one can approximate the Bessel function in the case of z << 1, by:

(A7) $$J_{0}\left(z\right)=\overset{\infty }{\underset{k=0}{\sum }}\frac{{\left(-1\right)}^{k}}{k!\left(0+k\right)!}{\left(\frac{z}{2}\right)}^{2k}=1+\ldots$$

(A8) $$J_{1}\left(z\right)=\overset{\infty }{\underset{k=0}{\sum }}\frac{{\left(-1\right)}^{k}}{k!\left(0+k\right)!}{\left(\frac{z}{2}\right)}^{2k+1}=\frac{z}{2}+\ldots$$

Then, the following expression is obtained:

(A9) $$i_{s}\left(t\right)=A_{i}\cos(\omega_{e}t)+AF\sin(\omega_{e}t)\cos(\omega_{m}t)+\varepsilon_{i}\left(t\right)$$

In conclusion:

(A10) $$i_{s}\left(t\right)=A_{i}\cos(\omega_{e}t)+\frac{A_{i}F}{2}\sin\left[\left(\omega_{e}+\omega_{m}\right)t\right]+\frac{A_{i}F}{2}\sin\left[\left(\omega_{e}-\omega_{m}\right)t\right]+\varepsilon_{i}\left(t\right)$$
